# Trps1 and Its Target Gene *Sox9* Regulate Epithelial Proliferation in the Developing Hair Follicle and Are Associated with Hypertrichosis

**DOI:** 10.1371/journal.pgen.1003002

**Published:** 2012-11-01

**Authors:** Katherine A. Fantauzzo, Mazen Kurban, Brynn Levy, Angela M. Christiano

**Affiliations:** 1 Department of Dermatology, Columbia University, New York, New York, United States of America; 2 Department of Genetics and Development, Columbia University, New York, New York, United States of America; 3 Department of Pathology and Cell Biology, Columbia University, New York, New York, United States of America; University of California San Diego, United States of America

## Abstract

Hereditary hypertrichoses are a group of hair overgrowth syndromes that are extremely rare in humans. We have previously demonstrated that a position effect on *TRPS1* is associated with hypertrichosis in humans and mice. To gain insight into the functional role of Trps1, we analyzed the late morphogenesis vibrissae phenotype of *Trps1^Δgt^* mutant mice, which is characterized by follicle degeneration after peg downgrowth has been initiated. We found that Trps1 directly represses expression of the hair follicle stem cell regulator *Sox9* to control proliferation of the follicle epithelium. Furthermore, we identified a copy number variation upstream of *SOX9* in a family with hypertrichosis that significantly decreases expression of the gene in the hair follicle, providing new insights into the long-range regulation of *SOX9*. Our findings uncover a novel transcriptional hierarchy that regulates epithelial proliferation in the developing hair follicle and contributes to the pathology of hypertrichosis.

## Author Summary

The various ectodermal appendages found in nature have evolved over time to allow organisms to better adapt to their environment. These include hair, feathers, scales, nails, teeth, beaks, horns, and a wide array of eccrine glands. The hair follicle is an ectodermal appendage unique to mammals that serves a wide array of functions, including thermoregulation, sensation, and communication. Hair follicle formation begins during embryogenesis through a series of interactions between adjacent epithelial and mesenchymal tissues. The mechanisms by which the diverse cells types of the hair follicle arise and the contribution of progenitor cells to the processes of growth and differentiation are not completely understood. Here, we have identified the transcription factor Trps1 as a novel regulator of epithelial proliferation in the developing hair follicle, through its control of *Sox9*, a gene known to regulate hair follicle stem cells. Moreover, we demonstrate that duplicated genetic material upstream of *SOX9*, which alters expression of the gene, results in a rare form of hereditary hair overgrowth syndrome in humans.

## Introduction

Hypertrichosis is defined as excessive hair growth for a particular site of the body or age of a patient that is not hormone-dependent. Hypertrichoses are characterized on the basis of multiple criteria: cause (genetic or acquired), age of onset, extent of hair distribution (universal or localized) and affected sites. Hereditary hypertrichoses are very rare in humans, affecting as few as one in one billion individuals [1]. Whereas many additional anomalies are associated with hypertrichosis, only a subset of disorders with congenital hypertrichosis present with excessive hair as the primary clinical feature. These include hypertrichosis universalis (OMIM 145700) [2], Ambras type (OMIM 145701) [3], X-linked hypertrichosis (OMIM 307150) [4] and generalized hypertrichosis terminalis with or without gingival hyperplasia (OMIM 135400) [5].

We previously demonstrated that a position effect on the zinc-finger transcription factor *TRPS1* is associated with two hypertrichosis models, Ambras syndrome (AS) in humans and the Koala phenotype in mice [6]. Consistent with a causative role for Trps1 in hypertrichosis, the protein is expressed in the nuclei of mesenchyme-derived dermal papilla cells and the proliferative epithelial cells of human and mouse hair follicles [7].

Heterozygous germline mutations in *TRPS1* on chromosome 8q23 in humans result in autosomal dominant inheritance of trichorhinophalangeal syndrome types I and III (TRPS1 I, OMIM 190350; TRPS III, OMIM 190351) [8], [9], which are characterized by sparse and slow-growing scalp hair, as well as craniofacial and skeletal abnormalities [10]. Correspondingly, homozygous mutant mice in which the GATA-type zinc-finger domain of Trps1 was deleted (*Trps1^Δgt/Δgt^*) were reported to have a number of hair follicle, craniofacial and skeletal defects that mirror the phenotypic characteristics of human TRPS patients [11]. *Trps1^Δgt/Δgt^* mice die within six hours of birth due to respiratory failure stemming from thoracic skeletal defects. Homozygous mutant mice were reported to completely lack vibrissae follicles during late gestation. In addition, neonatal *Trps1^Δgt/Δgt^* mice had an approximately 50 percent reduction in dorsal pelage follicle density compared to their wild-type littermates, whereas heterozygous mice had an intermediate pelage phenotype [11]. *Trps1^−/−^* null mice were subsequently generated and were similarly reported to display severe hair follicle abnormalities [12].

We recently performed a detailed histological analysis of early vibrissa follicle morphogenesis in *Trps1^Δgt/Δgt^* embryos from E12.5–E13.5 [13]. We found that the mutant vibrissae were reduced in number, irregularly spaced and developmentally delayed when compared to their wild-type counterparts [13]. Additional analyses revealed that these defects were likely due to disruption of Wnt signaling and the misexpression of several transcription factors and extracellular matrix proteins regulated by Trps1 in the mutant whisker pads [13]. While these studies collectively revealed a requirement for Trps1 during early vibrissa follicle formation, they did not address the mechanism(s) underlying the follicle degeneration observed later in these embryos.

Hypertrichosis had previously been reported in a case of partial trisomy 17q22-qter associated with a *de novo* unbalanced translocation [14], suggesting that the distal portion of human chromosome 17q may contain dosage-sensitive genes that contribute to excessive hair growth. Recently, a series of microdeletions were reported on chromosome 17q24.2–q24.3 in three cases of familial congenital generalized hypertrichosis terminalis with gingival hyperplasia (CGHT), as well as a *de novo* microduplication within this same region in a case of sporadic CGHT [15]. The minimal region common to each of these cases lies 2.5 Mb upstream of *SOX9*, a gene previously shown to be required for the specification and maintenance of hair follicle stem cells in mice [16], [17].

Here, we uncover a novel transcriptional hierarchy in the hair follicle in which Trps1 regulates *Sox9* to control epithelial proliferation in the developing vibrissa follicle in mice. Furthermore, we identify a copy number variation less than 1 Mb upstream of *SOX9* in a family with CGHT that significantly decreases expression of the gene in the hair follicle, providing significant insight into the pathology of human hypertrichosis.

## Results

### Late morphogenesis vibrissa follicle abnormalities in *Trps1^Δgt/Δgt^* mutant embryos

**Figure 1:**
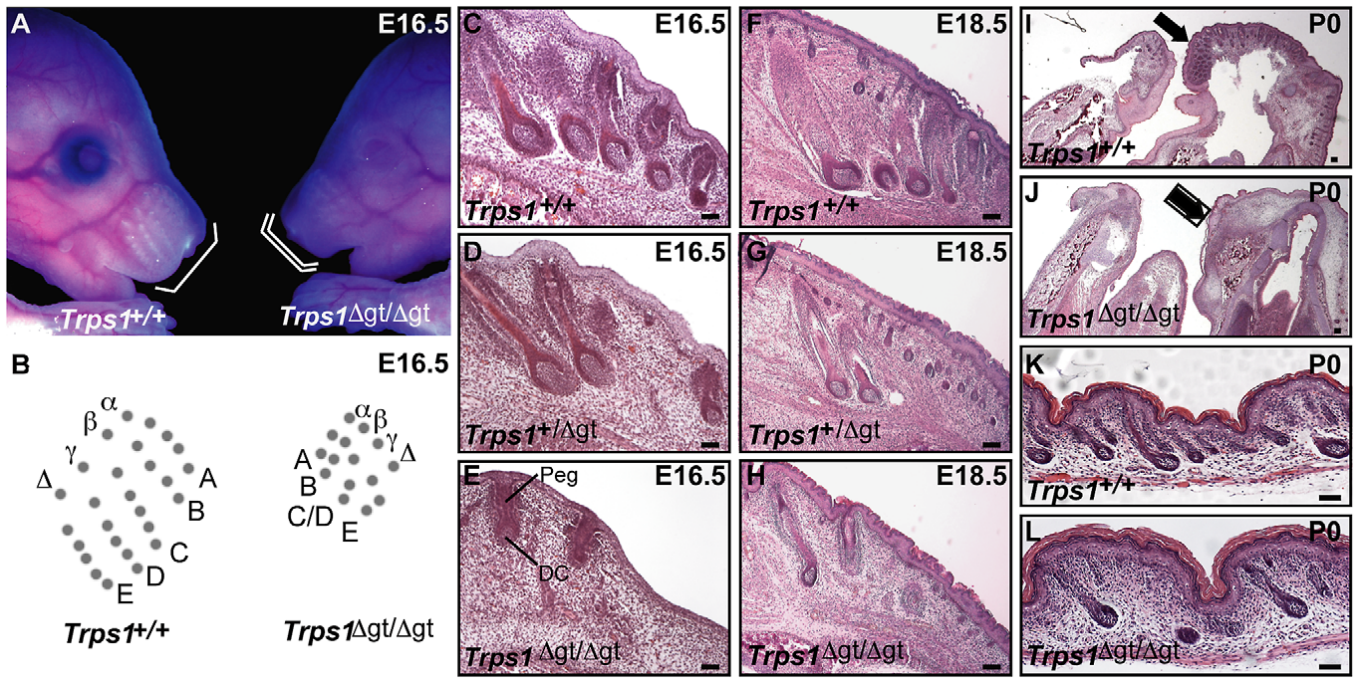
Late morphogenesis vibrissa follicle abnormalities in *Trps1^Δgt/Δgt^* embryos. (A) *Trps1^+/+^* and *Trps1^Δgt/Δgt^* embryos at E16.5. Note the reduced number of vibrissae follicles and decreased size of the maxillary region (white brackets) in mutant embryos. (B) *Trps1^+/+^* embryos have five rows of vibrissae (rows A–E), whereas *Trps1^Δgt/Δgt^* embryos have four rows due to a convergence of rows C and D. (C–H) Hematoxylin and eosin staining of transverse *Trps1^+/+^*, *Trps1^+/Δgt^* and *Trps1^Δgt/Δgt^* whisker pad sections at E16.5–E18.5 revealed small and irregularly spaced vibrissae follicles in heterozygous and homozygous mutant embryos. (I–L) Hematoxylin and eosin staining of sagittal *Trps1^+/+^* and *Trps1^Δgt/Δgt^* head and dorsal skin sections at P0. Note the absence of hair follicles in the upper and lower jaws (black arrows) (I,J) and reduction in pelage follicle density (K,L) in mutant mice. Peg, epithelial peg; DC, dermal condensate. Scale bars, 100 µm.

We began by performing a thorough histological analysis of vibrissa follicle morphogenesis during late gestation in *Trps1^Δgt/Δgt^* embryos. Similar to the defects observed during early morphogenesis in these embryos [13], the mutant vibrissae follicles that were present at E16.5 were reduced in number, irregularly spaced and smaller than wild-type vibrissae, with evidence of both an epithelial peg and dermal condensate (Figure 1A–1E). However, the development of these mutant vibrissae follicles was subsequently arrested, and they degenerated after peg downgrowth had been initiated so that they were rarely visible at birth (Figure 1F–1J). Interestingly, heterozygous *Trps1^+/Δgt^* embryos displayed an intermediate vibrissae phenotype (Figure 1D and 1G), with vibrissae follicles that were slightly larger, more advanced in development and greater in number than those detected in *Trps1^Δgt/Δgt^* mutant embryos (Figure 1E and 1H), indicating a dose-dependent requirement for Trps1 in multiple hair types. We additionally confirmed the reduction in pelage follicle density reported in homozygous mutant animals [11] (Figure 1K and 1L).

### *Trps1^Δgt/Δgt^* mutant vibrissae follicles exhibit increased levels of proliferation

**Figure 2:**
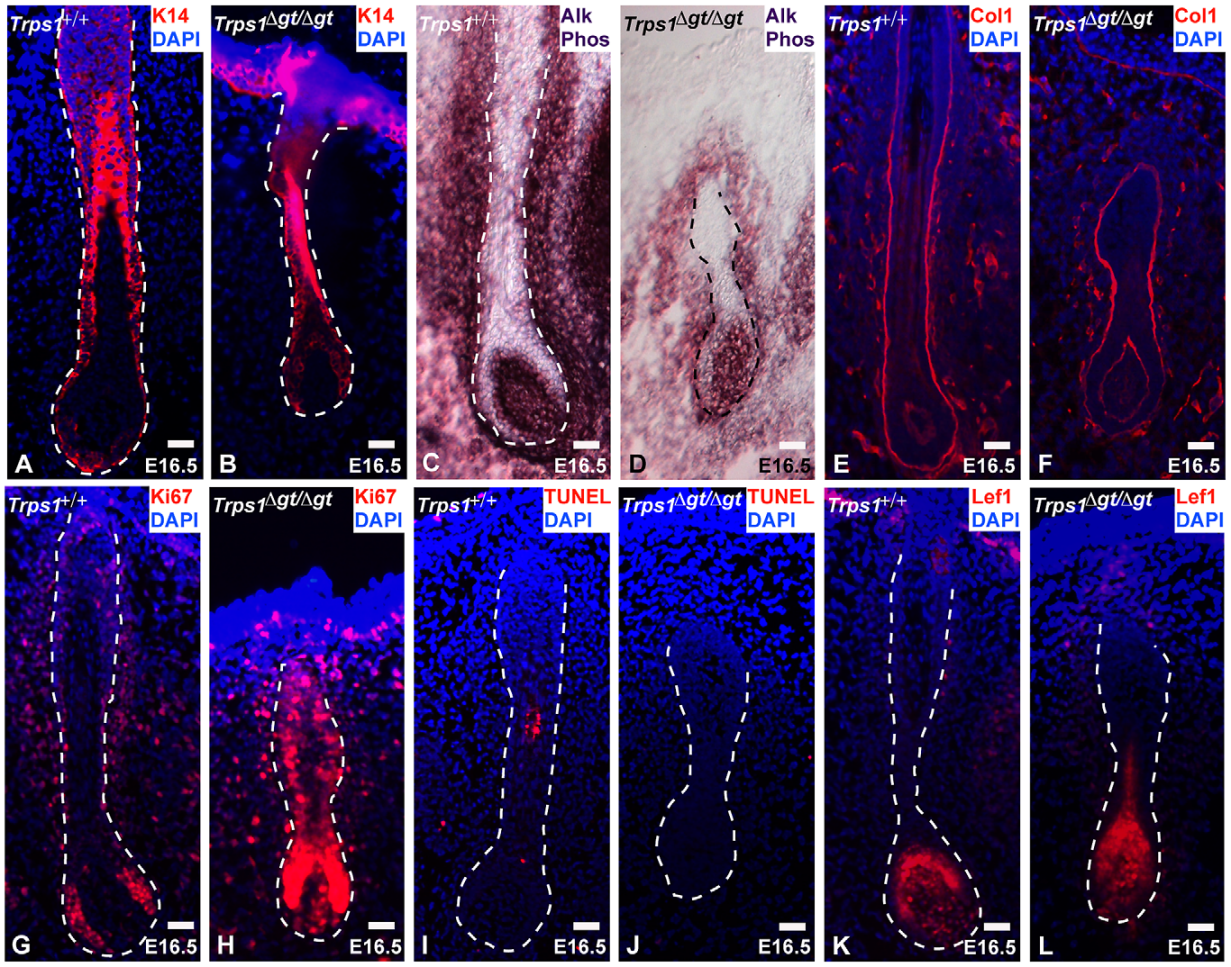
*Trps1^Δgt/Δgt^* vibrissae follicles exhibit increased levels of proliferation. (A,B) Keratin 14 staining (red) was consistent in the epithelial compartment of *Trps1^+/+^* and *Trps1^Δgt/Δgt^* vibrissae follicles at E16.5. (C,D) Alkaline phosphatase staining (purple) revealed an intact dermal papilla and reduced collagen capsule surrounding *Trps1^Δgt/Δgt^* mutant vibrissae. (E,F) Collagen type I staining (red) was consistent in the glassy membrane of *Trps1^+/+^* and *Trps1^Δgt/Δgt^* vibrissae follicles. (G,H) Ki67 staining (red) revealed a marked increase in proliferation throughout *Trps1^Δgt/Δgt^* mutant vibrissae follicles. (I,J) TUNEL staining (red) indicated similar levels of apoptosis between *Trps1^+/+^* and *Trps1^Δgt/Δgt^* vibrissae. (K,L) Lef1 staining (red) was consistent in the dermal papillae and matrix cells of *Trps1^+/+^* and *Trps1^Δgt/Δgt^* vibrissae follicles. Nuclei were stained with DAPI (blue). Scale bars, 100 µm.

To assess the mechanisms underlying vibrissa follicle degeneration in *Trps1^Δgt/Δgt^* mutant embryos, we examined the levels of proliferation and apoptosis in these follicles, as well as the expression of numerous cell-type specific markers at embryonic day 16.5 (E16.5). Immunofluorescence analyses revealed consistent keratin 14 (K14) expression in the epithelial compartments of wild-type and mutant vibrissae follicles (Figure 2A and 2B), and alkaline phosphatase staining displayed an intact dermal papilla, but a considerably smaller and less dense collage capsule surrounding *Trps1^Δgt/Δgt^* mutant vibrissae (Figure 2C and 2D). Collagen type I expression was comparable in the glassy (basement) membranes of wild-type and *Trps1^Δgt/Δgt^* vibrissae follicles (Figure 2E and 2F). Immunofluorescence analyses of Ki67 expression revealed a marked increase in proliferation throughout the developing *Trps1^Δgt/Δgt^* vibrissa follicle (Figure 2G and 2H), while the TUNEL assay indicated similar levels of apoptosis between the two follicle types (Figure 2I and 2J). Of note, expression of the Wnt effector Lef1 was consistent in the dermal papillae and matrix cells of *Trps1^+/+^* and *Trps1^Δgt/Δgt^* vibrissae follicles at this timepoint (Figure 2K and 2L), indicating that the deregulation of the canonical Wnt pathway detected in *Trps1^Δgt/Δgt^* vibrissae placodes at E12.5 [13] is no longer observed during later morphogenesis.

### Trps1 directly represses the expression of *Sox9* in the vibrissa follicle

Having previously demonstrated that Trps1 directly regulates the expression of the bulge stem cell compartment markers *Lhx2* and *Tnc* in the murine whisker pad [13], we next asked whether Trps1 might also regulate *Sox9* in the hair follicle. As mentioned above, Sox9 crucially regulates several aspects of hair follicle stem cell activity in mice [16], [17] and lies near the minimal region common to several cases of CGHT [15]. We began by performing immunofluorescence analysis examining the expression of Sox9 during vibrissa follicle morphogenesis. At E12.5, Sox9 was expressed throughout the whisker pad epidermis, with increased expression in the suprabasal layers of the epithelial placode (Figure S1A). By the peg stage at E14.5, Sox9 was expressed throughout the epithelial compartment of the downgrowing follicle, with the exception of the matrix cells (Figure S1B). From E16.5–E18.5, Sox9 continued to be expressed throughout the follicle epithelium, with increased expression in the matrix, inner root sheath and outer root sheath layers (Figure S1C and S1D). By P0 however, Sox9 expression became noticeably restricted to the outer root sheath cells extending along the length of the follicle (Figure S1E and S1E′). Interestingly, faint Sox9 expression was also detected in the dermal papilla as early as E14.5 (Figure S1B, S1C′, S1D′ and S1E″), as well as in the dermal cells of the collagen capsule surrounding the developing vibrissae follicles (Figure S1B–S1E). With few exceptions, this pattern of Sox9 staining in the vibrissa follicle is consistent with that reported for the developing pelage follicle [16], [17], and also with the expression pattern of Trps1 in developing vibrissae [6].

**Figure 3:**
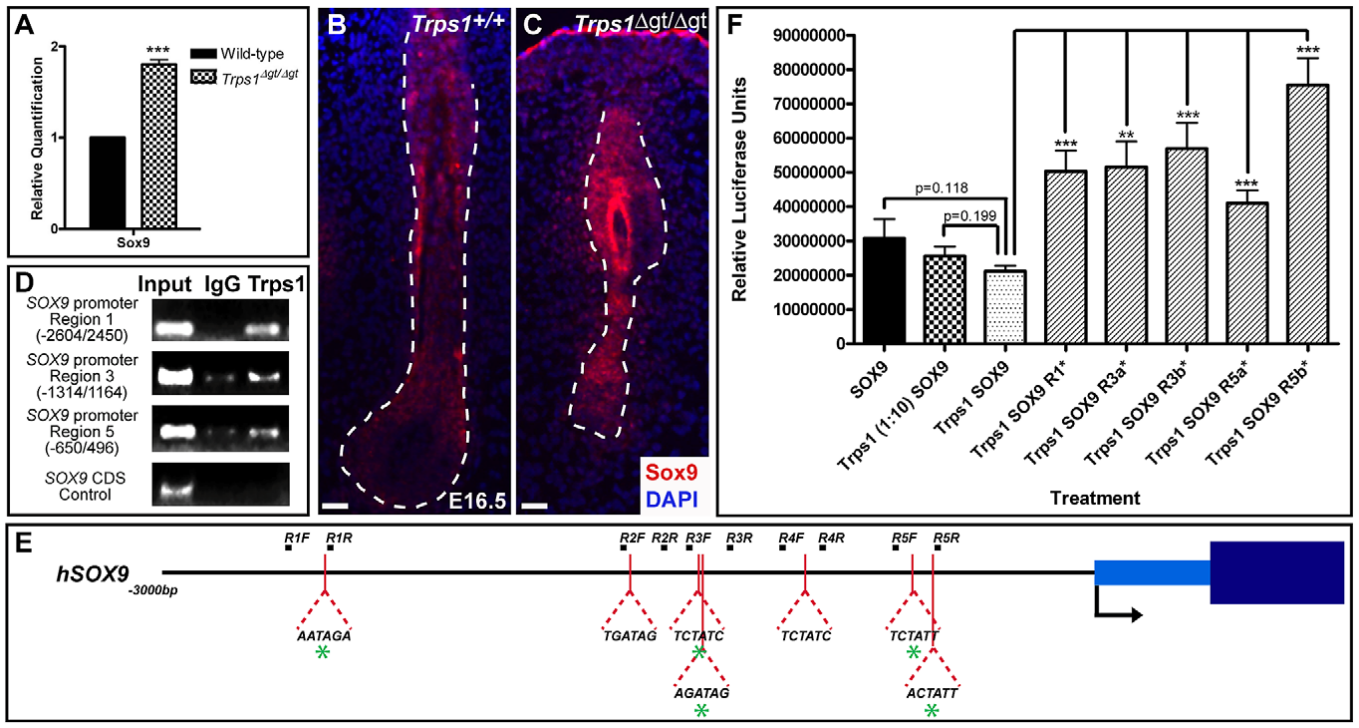
Trps1 directly represses the expression of *Sox9* in the vibrissa follicle. (A) Bar graph depicting quantitative RT-PCR values revealing increased expression of *Sox9* in E12.5 *Trps1^Δgt/Δgt^* whisker pad samples as compared to wild-type expression levels. Data are represented as mean ± standard deviation. *** = p<0.001. (B,C) Immunofluorescence analyses revealed increased Sox9 expression (red) throughout the epithelial compartment of *Trps1^Δgt/Δgt^* vibrissae follicles and surrounding collagen capsule at E16.5. Nuclei were stained with DAPI (blue). Scale bars, 100 µm. (D) Trps1 bound up to five sites in the *SOX9* promoter in endogenous chromatin immunoprecipitation experiments in HEK 293T cells. No binding was observed in a coding sequence negative control region. (E) Identification of canonical GATA-binding sites within 3 kb of the transcriptional start site of *SOX9*. Green asterisks represent sites to which Trps1 was shown to bind. (F) Bar graph depicting relative luciferase units in luciferase reporter promoter assays demonstrating dose-dependent repression of *SOX9* transcription by Trps1. Mutation of each of the Trps1-binding sites from WGATAR to WGATCR (*) alleviated Trps1-mediated repression of *SOX9*. ** = p<0.01; *** = p<0.001.

We next performed qRT-PCR analysis comparing the expression of *Sox9* in wild-type versus *Trps1^Δgt/Δgt^* whisker pad samples at E12.5, when the vibrissae placodes are initially discernible and Sox9 expression is first observed. We found that *Sox9* was upregulated 1.80-fold (±0.05; p<0.001) in the mutant samples compared to wild-type expression levels (Figure 3A). Furthermore, immunofluorescence analyses at E16.5 demonstrated continued, increased Sox9 protein expression throughout the epithelial compartment and surrounding collagen capsule of *Trps1^Δgt/Δgt^* vibrissae follicles (Figure 3B and 3C).

To further dissect the relationship between Trps1 and Sox9, we performed endogenous chromatin immunoprecipitation experiments in HEK 293T cells to determine whether Trps1 can directly bind the *SOX9* promoter. TRPS1 has previously been shown to specifically bind the consensus GATA sequence (WGATAR) in DNA [18], [19]. Upon identifying seven consensus GATA-binding sites within 3 kb of the transcriptional start site of *SOX9* (Figure 3E), we found that Trps1 bound up to five of these sites in the *SOX9* promoter (Figure 3D). We next performed luciferase reporter promoter assays in HEK 293T cells and demonstrated that Trps1 represses *SOX9* transcription (31.11±5.44%; p = 0.118) in a dose-dependent manner (Figure 3F). This repression was alleviated upon mutation of each of the Trps1-binding sites from WGATAR to WGATCR (p<0.01; Figure 3F).

### A *Shh* null allele can rescue the vibrissae phenotype of *Trps1^+/Δgt^* embryos

Decreased Sox9 expression was previously observed in both *Shh^−/−^* and *Gli2^−/−^* mutant hair germs [17], indicating that Shh signaling may also regulate Sox9 expression in the hair follicle. This relationship is supported by numerous reports that implicate SHH activation of Sox9 expression during chondrogenesis in chick [20], mouse [21], [22] and humans [23]. To determine whether Trps1 colocalizes with cells expressing Shh in the vibrissa follicle, we performed immunofluorescence analyses on serial sections of adult *Shh^Ires-nLacZ^* vibrissae follicles, wherein nuclear β-galactosidase staining is observed in cells expressing Shh. We demonstrated that Trps1 colocalizes with β-galactosidase in cells of the matrix and inner root sheath layers, indicating coexpression of Trps1 and Shh in these proliferative cells at the base of the follicle (Figure S2).

**Figure 4:**
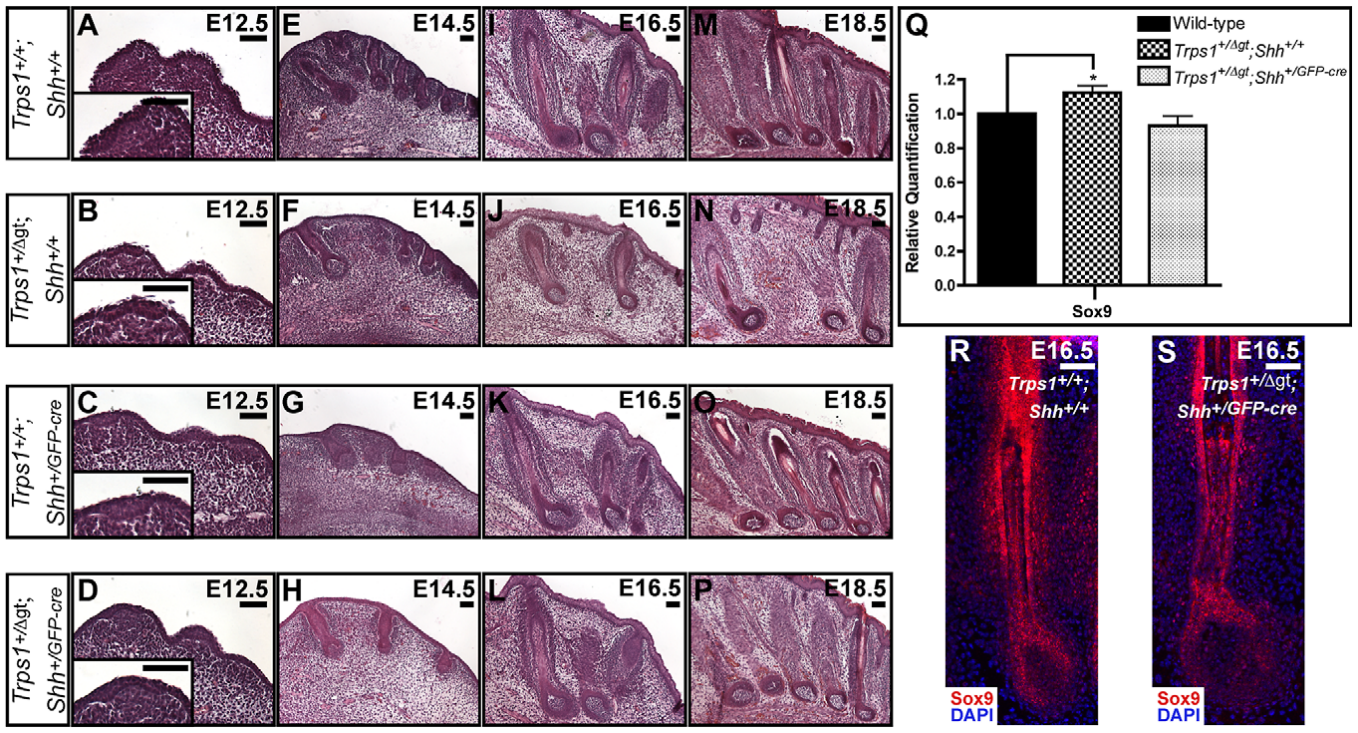
A *Shh* null allele can rescue the vibrissae phenotype of *Trps1^+/Δgt^* embryos. (A–P) Hematoxylin and eosin staining of transverse *Trps1^+/+^;Shh^+/+^*, *Trps1^+/Δgt^;Shh^+/+^*, *Trps1^+/+^;Shh^+/GFP-cre^*, and *Trps1^+/Δgt^;Shh^+/GFP-cre^* whisker pad sections at E12.5–E18.5 revealed that *Trps1^+/+^;Shh^+/GFP-cre^* vibrissae (C,G,K,O) developed similarly to wild-type follicles (A,E,I,M), while *Trps1^+/Δgt^;Shh^+/+^* embryos exhibited a reduction in vibrissae placode number at E12.5 (B) and displayed follicles that were reduced in number, irregularly spaced and slightly smaller than wild-type vibrissae throughout the remainder of embryogenesis (F,J,N). The vibrissae phenotype of *Trps1^+/Δgt^;Shh^+/+^* embryos was completely rescued at all timepoints in *Trps1^+/Δgt^;Shh^+/GFP-cre^* compound heterozygous mice (D,H,L,P). (Q) Bar graph depicting quantitative RT-PCR values revealing increased expression of *Sox9* in E16.5 *Trps1^+/Δgt^;Shh^+/+^* whisker pad samples as compared to wild-type expression levels. *Sox9* expression was recovered to wild-type levels in *Trps1^+/Δgt^;Shh^+/GFP-cre^* whisker pad samples. Data are represented as mean ± standard deviation. * = p<0.05. (R,S) Sox9 expression (red) was recovered to wild-type levels throughout the epithelial compartment of the vibrissae follicles in *Trps1^+/Δgt^;Shh^+/GFP-cre^* embryos as detected by immunofluorescence analyses. Nuclei were stained with DAPI (blue). Scale bars, 100 µm.

Postulating that Shh signaling and Trps1 expression would have opposing effects on *Sox9* expression in the hair follicle, we next asked whether introduction of a *Shh* null allele could rescue the vibrissae phenotype of *Trps1^+/Δgt^* embryos. We generated *Trps1^+/Δgt^;Shh^+/GFP-cre^* compound heterozygous mice and performed detailed histological examinations of their vibrissae follicles at multiple timepoints throughout embryogenesis. Transverse whisker pad sections revealed that *Trps1^+/+^;Shh^+/GFP-cre^* vibrissae (Figure 4C, 4G, 4K and 4O) developed similarly to wild-type follicles from E12.5–E18.5 (Figure 4A, 4E, 4I and 4M). As expected, *Trps1^+/Δgt^;Shh^+/+^* embryos exhibited a reduction in vibrissae placode number at E12.5 (Figure 4B), and displayed follicles that were reduced in number, irregularly spaced and slightly smaller than wild-type vibrissae throughout the remainder of embryogenesis (Figure 4F, 4J and 4N). However, this phenotype was completely rescued at all timepoints in *Trps1^+/Δgt^;Shh^+/GFP-cre^* compound heterozygous mice (Figure 4D, 4H, 4L and 4P). Importantly, the expression of *Sox9* transcripts returned to wild-type levels in compound heterozygous whisker pads (Figure 4Q) and Sox9 protein expression was restored to wild-type levels throughout the epithelial compartment of the vibrissae follicles in *Trps1^+/Δgt^;Shh^+/GFP-cre^* embryos (Figure 4R and 4S).

### Copy number variations upstream of *SOX9* associated with hypertrichosis

**Figure 5:**
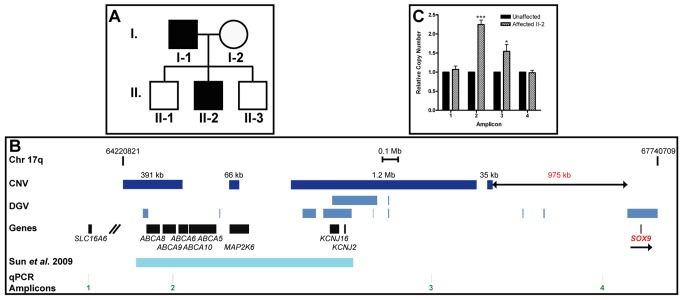
Copy number variations upstream of *SOX9* associated with hypertrichosis. (A) Pedigree of family in which the father (patient I-1) and proband (patient II-2) exhibited CGHT. (B) Map of human chromosome 17q spanning base pairs 64,220,821–67,740,709 according to build hg18. Copy number variations (CNV) detected in our analyses are represented by dark blue boxes. The telomeric end of the duplication region identified here lies 975 kb upstream of *SOX9*. Duplications present in the Database of Genomic Variations (DGV) or previously identified by Sun *et al.* [15] are represented by lighter blue boxes. The locations of amplicons used in quantitative PCR analysis are represented by green lines. Scale bar, 0.1 Mb. (C) Bar graph depicting quantitative PCR values revealing significant increases in relative copy number of two amplicons in the proband within the duplication region identified here as compared to an unaffected control individual. Data are represented as mean ± standard deviation. * = p<0.05; *** = p<0.001.

We previously demonstrated that a position effect on *TRPS1* is associated with cases of hypertrichosis in both humans and mice [6]. Here, we report a family in which the father (patient I-1) and son (patient II-2) exhibited CGHT with mild gingival hyperplasia (Figure 5A). The father is of French and African descent, while the mother is of German descent. The affected patients presented with striking generalized hypertrichosis, which was most prominent on the face, ears and upper trunk. Both father and son displayed bushy eyebrows with synophrys and elongated eyelashes, as well as downslanted fissures and epicanthic folds. The hair covering the face and body was often coarse, straight and black. The parents reported that the son developed progressive hypertrichosis shortly after birth. Both patients additionally exhibited bulbous nasal tips, thick nasal wings, a long, prominent philtrum with a deep groove and mild thoracic kyphoscoliosis. No lip swelling or eversion was observed. Endocrine and metabolic assessments were unremarkable for both patients.

To search for chromosomal anomalies in these patients, genomic DNA was isolated from peripheral blood samples collected from the family members and genotyped using the Affymetrix Cytogenetics Whole-Genome 2.7 M Array and Affymetrix Genome-Wide Human SNP Array 6.0 for Cytogenetics. The resulting data were analyzed with the Affymetrix Chromosome Analysis Suite Version 1.0.1 software. We obtained consistent results with both arrays, identifying a series of four novel duplications with sizes of 391 kb, 66 kb, 1.2 Mb and 35 kb, respectively, within a 2.4 Mb region in chromosome 17q24.2–q24.3 in both affected patients. The telomeric end of this region lies 975 kb upstream of the Trps1 target gene *SOX9* (Figure 5B). While eight duplications with a combined size of 487 kb are reported in this region in the public Database of Genomic Variants, our findings uncover approximately 1.2 Mb of novel duplicated chromosomal material within this region. Furthermore, the duplicated region encompasses the 1.4 Mb duplication identified in a sporadic case of CGHT reported by Sun *et al.* [15], and extends 86 kb beyond the centromeric border and 917 kb beyond the telomeric border of that region (Figure 5B).

To confirm the duplications, we performed quantitative PCR analysis using the DNA of patient II-2, the proband, as well as that of an unaffected control individual, to examine the relative copy number of amplicons across the region. Patient II-2 had a 2.24-fold increase (±0.12; p<0.001) in relative copy number of one amplicon within the region (amplicon 2), and a 1.54-fold increase (±0.18; p<0.05) of a second amplicon within the duplication region (amplicon 3) as compared to an unaffected control individual. There were no significant changes in relative copy number of two amplicons (amplicons 1 and 4) on either side of the duplication region in patient II-2 (Figure 5C).

We next performed fluorescent *in situ* hybridization (FISH) analysis to determine the orientation of the large 1.2 Mb duplication within our candidate region. Interphase chromosome spreads prepared from the blood of patient II-2 were hybridized with green 5-Fluorescein dUTP labeled probe RP11 clone 164B17 and orange 5-TAMRA dUTP labeled probe RP11 clone 831L20, which span chromosome 17q base pairs 65,334,626–65,500,838 and 66,328,117–66,543,177, respectively. FISH analysis revealed one wild-type chromosome with a single hybridization signal for each probe in the patient cells, as well as one chromosome containing duplicated genetic material with two hybridization signals for each probe (Figure S3). The pattern of probe hybridization in the rearranged chromosome (green-orange-orange-green) demonstrated that the 1.2 Mb duplication in patient II-2 was an inverted duplication (Figure S3).

**Figure 6:**
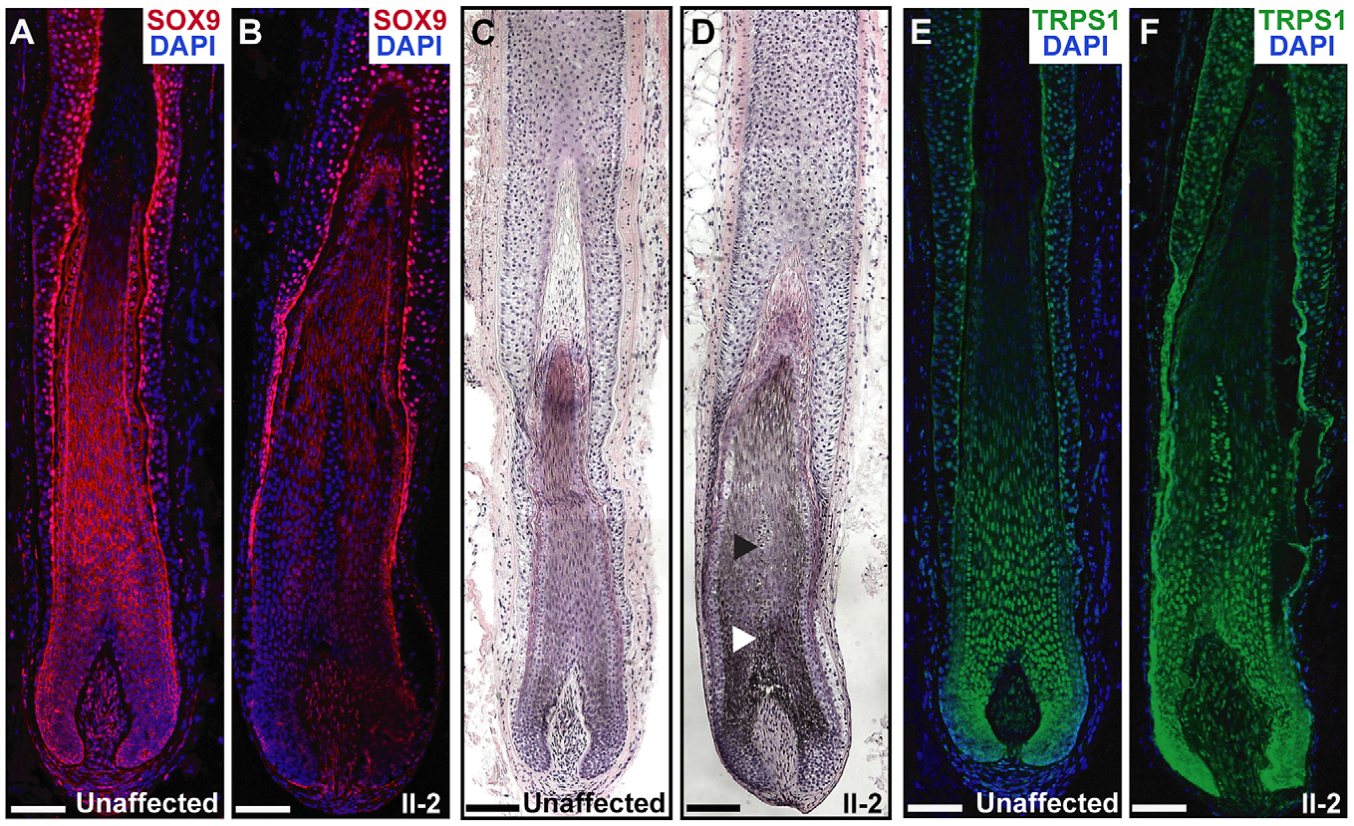
Expression and histological analyses of CGHT follicles. (A,B) SOX9 expression (red) was decreased in the hair follicle of the proband (B) as compared to an unaffected control follicle (A), particularly in the proliferative epithelial cells at the base of the follicle, as detected by immunofluorescence analyses. Nuclei were stained with DAPI (blue). (C,D) Hematoxylin and eosin staining of hair follicles from an unaffected control individual (C) and patient II-2 (D), revealing increased pigmentation and diameter in the CGHT follicle, particularly in the medulla layer (arrowheads) at the center of the hair shaft. (E,F) Consistent TRPS1 expression (green) between control (E) and CGHT follicles (F). Nuclei were stained with DAPI (blue). Scale bars, 100 µm.

To determine the effect of the duplications in the proband on SOX9 expression in the skin and hair follicle, we performed immunofluorescence analyses on a biopsy taken from the posterior neck of patient II-2 and a sample taken from the lower scalp of an unaffected control individual. Patient II-2 had a striking decrease in SOX9 protein expression throughout the follicle epithelium as compared to normal expression levels, primarily in the proliferative epithelial cells at the base of the follicle (Figure 6A and 6B). Histological analyses demonstrated that the patient follicles were more highly pigmented than those of the unaffected control individual, and larger in diameter, particularly in the medulla layer in the center of the hair shaft (Figure 6C and 6D). TRPS1 expression was similar within the follicles of patient II-2 and the control individual (Figure 6E and 6F), consistent with its role upstream of *SOX9* expression. In conclusion, these results demonstrate that a large 2.4 Mb duplication 975 kb upstream of *SOX9* significantly decreases the expression of the gene in the hair follicle, consistent with a position effect on *SOX9*.

## Discussion

While human hypertrichoses have been described in literature dating back to the 16^th^ century, the genetic determinants and molecular mechanisms underlying these conditions have remained elusive. We have previously demonstrated that a position effect on *TRPS1* is associated with hypertrichosis in both humans and mice [6], providing the first evidence for a position effect associated with abnormalities in hair follicle development. Here, we establish that a position effect on the Trps1 target gene *SOX9* is likely involved in the pathology of human hypertrichosis. Our findings provide the first instance of direct upstream regulation of the hair follicle stem cell specification gene *Sox9*, revealing its role in regulating epithelial proliferation downstream of both Trps1 and the Shh pathway in the developing follicle.

Our data indicate that Trps1 expression and Shh signaling balance the regulation of *Sox9* expression in proliferative hair follicle epithelial cells, with Shh and its downstream effector(s) acting as positive regulators of *Sox9* expression and Trps1 repressing *Sox9* transcription. Gli2 is the main mediator of Shh signaling in the skin and hair follicle [24] and ectopic overexpression of a constitutively active form of Gli2, *ΔNGli2*, in the basal layer of the skin is sufficient to induce *Sox9* expression, suggestive of direct activation of *Sox9* expression by Gli2 [17]. We did not identify either of the reported consensus GLI binding sites [25], [26] within the 3 kb *SOX9* promoter that we analyzed, indicating that Trps1 may regulate *SOX9* expression at distinct sites from Gli proteins. Consistent with our model, we demonstrated that a *Shh* null allele is able to completely rescue the vibrissae phenotype of *Trps1^+/Δgt^* embryos in compound heterozygous mice and restore Sox9 expression to wild-type levels.

Consistent with a downstream convergence of Trps1 and Shh pathway signaling in the hair follicle, *Trps1^Δgt/Δgt^* follicles share a number of phenotypic similarities with *Shh^−/−^* and *Gli2^−/−^* mutant follicles, most notably a reduction in follicle number and follicle arrest shortly after induction [24], [27], [28]. While *Shh^−/−^* embryos were reported to develop vibrissae follicles despite their extensive craniofacial defects, *Gli2^−/−^* mice had fewer and under-developed vibrissae [24]. Furthermore, the number of pelage follicles in *Shh^−/−^* and *Gli2^−/−^* mutant mice is reduced by 25 to 60 percent. The pelage follicles that do form have small hair germs that arrest shortly after induction, with evidence of both epithelial invasion of the dermis and dermal condensation of the mesenchyme at the base of the germ [24], [27], similar to the phenotype observed in *Trps1^Δgt/Δgt^* mutant embryos. When grafted onto immunocompromised *nude* mice, whole embryonic dorsal *Shh^−/−^* skin exhibited increased proliferation in the follicle epithelium [27], [28], comparable to the increased proliferation observed throughout *Trps1^Δgt/Δgt^* vibrissae follicles.

Conditional ablation of *Sox9* in the epidermal compartment of the skin and hair follicle during embryogenesis (*K14-Cre;Sox9^fl/fl^*) resulted in an 80 percent reduction in vibrissae follicle number at birth and the absence of an external pelage hair coat as early as P6 [16], akin to the sparse vibrissae and pelage observed in *Trps1^Δgt/Δgt^* mice. Similarly, postnatal ablation of *Sox9* in the skin epithelia (*Y10-Cre;Sox9^fl/fl^*) resulted in small, atrophic pelage hairs in the caudal region of the body, many of which degenerated after the first hair cycle [17], pointing to a requirement for Sox9 in maintenance of the hair follicle after early development. Furthermore, both homozygous mutant *Y10-Cre;Sox9^fl/fl^* and *K14-Cre;Sox9^fl/fl^* mice exhibited a decrease in the number of proliferative matrix cells [16], [17]. As our results indicate that Trps1 represses *Sox9* expression, this reduced proliferation is analogous to the opposite phenotype of increased proliferation throughout *Trps1^Δgt/Δgt^* vibrissae.

Shh is a morphogen that plays a key role in regulating the proliferation and downgrowth of the follicular epithelium during late morphogenesis [27]–[29], and in promoting anagen initiation during postnatal hair follicle cycling [30], [31]. Transient, ectopic expression of Shh in the dorsal skin can initiate anagen in resting telogen follicles and accelerate hair growth [30]. Notably, excessive activation of the Shh signaling pathway is a common feature of many hair follicle tumors, including basal cell carcinomas (BCC) [32]–[34]. Overexpression of *Shh*, *Gli1*, *Gli2* or an activated mutant allele of *Smo* in the murine epidermis was sufficient to induce BCC formation [32], [34]–[36], further supporting a role for the Shh pathway in regulating cell proliferation in the epithelia of the hair follicle. Sox9 expression is upregulated in both mouse and human BCC tumors [17] and was later shown to be a general marker of human BCC and additional hair follicle-derived tumors [37], consistent with its activation downstream of Shh signaling.

We found that Trps1 colocalizes with cells expressing Shh in the matrix and inner root sheath layers of the vibrissa follicle, and furthermore, that Sox9 is also expressed in these cells beginning at mid-morphogenesis. The highly proliferative matrix epithelial cells at the base of the follicle give rise to the various differentiating layers of the inner root sheath during hair follicle morphogenesis [38]. Postnatally, interactions between the mesenchyme-derived dermal papilla and the epithelial matrix cells similarly result in growth of the hair shaft during anagen [39]. The coexpression of Trps1, Shh and Sox9 in the matrix cells and their inner root sheath derivatives suggests a role for Trps1 in regulating vibrissa follicle proliferation during epithelial growth through its direct regulation of *Sox9*.

Sox9 has previously been shown to be required for specification of hair follicle stem cells [16]. Furthermore, genetic marking techniques have demonstrated that Sox9-derived progeny give rise to all the epithelial cells of the hair follicle [16]. We propose that the dysregulation of Sox9 in the absence of Trps1 would result in a defect in progenitor cell activity in the hair follicle. Upon increased Sox9 expression in *Trps1^Δgt/Δgt^* mutant vibrissae, additional hair follicle progenitor cells would be specified. However, in the absence of Sox9 repression by Trps1, these cells would proliferate prematurely, thereby depleting the follicle of slow-cycling progenitor cells with long-term proliferative potential. Consistent with this hypothesis, *Trps1^Δgt/Δgt^* embryos exhibit increased proliferation throughout vibrissae follicles prior to their degeneration. In the absence of progenitor cells to fuel the epithelial proliferation necessary to complete morphogenesis, these *Trps1^Δgt/Δgt^* follicles arrest. Lending support to this notion, conditional ablation of *Smo* in the hair follicle epithelium (*K14-Cre;Smo^fl/fl^*) resulted in decreased Shh signaling and *Sox9* expression in these cells. Importantly, these changes were accompanied by reduced proliferation in the matrix and depletion of the hair follicle stem cell niche [40].

A number of mutations in and around the human *SOX9* gene result in diseases with phenotypic similarities to TRPS types I and III. Notably, the two patients with CGHT reported here share many phenotypic similarities with AS patients, including hypertrichosis of the face, ears and upper trunk, a bulbous tip of the nose, thick nasal wings and a long, prominent philtrum with a deep groove [3], providing further evidence that SOX9 and TRPS1 function in the same developmental pathway.

Position effects have previously been described for a number of Trps1 target genes, including *SOX9* [41], [42], which lead to rare genetic skeletal disorders. Taken together, our data implicate that position effects on *TRPS1* as well as its target gene *SOX9* may play a causative role in human hypertrichoses. Thus, while intragenic mutations or deletions of each of these genes result in hair and bone abnormalities, they are also subject to long-range regulation that, upon disruption, can generate unique phenotypes at sites of the body where these genes are expressed.

## Materials and Methods

### Ethics statement

Upon obtaining informed consent, peripheral blood samples were collected from family members under approval of the Institutional Review Board of Columbia University and in adherence to the Declaration of Helsinki Principles. All mouse experiments were performed under approval of the Institutional Animal Care and Use Committee of Columbia University.

### Mice

*Trps1^+/tm1Shiv^* mice [11], referred to in the text as *Trps1^+/Δgt^*, were a generous gift of Dr. Ramesh Shivdasani, Dana-Farber Cancer Institute, Harvard Medical School. *Shh^+/tm4Amc^* mice [43], [44], referred to in the text as *Shh^+/Ires-nLacZ^*, were a generous gift of Dr. Ed Laufer, Columbia University. *Shh^+/tm1(EGFP/cre)Cjt^* mice [45], referred to in the text as *Shh^+/GFP-cre^*, were obtained from The Jackson Laboratory. Heterozygous *Shh^+/tm1(EGFP/cre)Cjt^* males were bred to heterozygous *Trps1^+/tm1Shiv^* females to generate compound heterozygous mice.

### Histology

Whole mouse muzzle skin and/or whole mouse dorsal skin was dissected at multiple timepoints from E12.5 (12.5 days post coitus, day of plug considered 0.5 days) through P0 (postnatal day 0) in 1× phosphate-buffered saline (PBS), fixed in 10% formalin for up to 72 hrs, washed through an ethanol series and embedded in paraffin. After deparaffinization and rehydration, 8 µm sections were stained with hematoxylin and eosin and permanently mounted with Permount (Thermo Fisher Scientific, Inc., Waltham, MA, USA) for observation under a light microscope. Sections were photographed using an HRC Axiocam fitted onto an Axioplan2 fluorescence microscope (Carl Zeiss, Inc., Thornwood, NY, USA). Sections of patient skin biopsies mounted in O.C.T. compound (Sakura Finetek USA, Inc., Torrance, CA, USA) and frozen in liquid nitrogen were similarly stained and photographed.

### Immunofluorescence analysis

Sections of whole mouse muzzle skin or patient skin biopsies mounted in O.C.T. compound (Sakura Finetek USA, Inc.) and frozen in liquid nitrogen were fixed in 4% paraformaldehyde (PFA)/0.1% Triton-X for 10 min at room temperature or in methanol for 15 min at −20°C followed by acetone for 2 min at −20°C and washed in PBS. The sections were then blocked for 1 hr in 10% heat-inactivated goat serum in PBS and incubated overnight at 4°C in primary antibody diluted in 1% serum in PBS. Primary antibodies and dilutions were as follows: anti-keratin 14 (1∶5,000; gift of Dr. Jurgen Schweizer, German Cancer Research Center); anti-Collagen type I (1∶500; Developmental Studies Hybridoma Bank); anti-Ki67 (1∶1,000; Abcam Inc., Cambridge, MA, USA); anti-Lef1 (1∶25; Santa Cruz Biotechnology, Santa Cruz, CA, USA); anti-Sox9 (1∶1,000; Santa Cruz Biotechnology); anti-Trps1 (1∶5,000; gift of Dr. Ramesh Shivdasani, Dana-Farber Cancer Institute, Harvard Medical School; [18]); anti-β-galactosidase (1∶1,000; MP Biomedicals, Solon, OH, USA). After washing in PBS, the sections were incubated in either an Alexa Fluor 594 goat anti-rabbit IgG or Alexa Fluor 488 goat anti-rabbit IgG (Molecular Probes, Invitrogen, Carlsbad, CA, USA) secondary antibody (1∶500) diluted in 1% serum in PBS for 1 hr. Sections were mounted in VECTASHIELD mounting medium with DAPI (Vector Laboratories, Burlingame, CA, USA) and photographed using an HRC Axiocam fitted onto an Axioplan2 fluorescence microscope (Carl Zeiss, Inc.) or an LSM 5 laser scanning Axio Observer Z1 confocal microscope (Carl Zeiss, Inc.).

### Detection of alkaline phosphatase activity

Alkaline phosphatase activity was detected based on a previously published protocol [46]. Briefly, 8 µm sections of E16.5 whole mouse muzzle skin mounted in O.C.T. compound (Sakura Finetek USA, Inc.) and frozen in liquid nitrogen were fixed in acetone at −20°C for 5 min and washed in PBS for 5 min at room temperature. The sections were then washed in buffer containing 0.1 M Tris-HCl pH 9.5 and 0.1 M NaCl for 5 min at room temperature and incubated in substrate containing 250 µg/mL 4-Nitro blue tetrazolium chloride (NBT; Roche Applied Science, Indianapolis, IN, USA) and 125 µg/mL 4-toluidine salt (BCIP; Roche Applied Science) diluted in the above buffer for 12 min in the dark. After a 5 min wash in PBS, sections were mounted in VECTASHIELD mounting medium for fluorescence (Vector Laboratories) and photographed using an HRC Axiocam fitted onto an Axioplan2 fluorescence microscope (Carl Zeiss, Inc.).

### TUNEL assay

TUNEL staining was performed on 8 µm sections of E16.5 whole mouse muzzle skin mounted in O.C.T. compound (Sakura Finetek USA, Inc.) and frozen in liquid nitrogen. Sections were fixed in 1% PFA/PBS for 10 min, washed in PBS and post-fixed in 2∶1 ethanol∶acetic acid for 5 min at −20°C before being fluorescently stained using the ApopTag Plus Fluorescein In Situ Apoptosis Detection Kit (Millipore, Billerica, MA, USA) according to the manufacturer's instructions. Sections were mounted in VECTASHIELD mounting medium with DAPI (Vector Laboratories) and photographed using an HRC Axiocam fitted onto an Axioplan2 fluorescence microscope (Carl Zeiss, Inc.). All positive signals were confirmed by DAPI staining.

### Quantitative RT–PCR

Total RNA was isolated from whole mouse muzzle skin at E12.5 or E16.5 using the RNeasy Mini Kit (Qiagen Inc., Valencia, CA, USA) according to the manufacturer's instructions. First-strand cDNA was synthesized using a ratio of 2∶1 random primers: Oligo (dT) primer and SuperScript III RT (Invitrogen) according to the manufacturer's instructions. qRT-PCR was performed on an ABI 7300 machine and analyzed with ABI Relative Quantification Study software (Applied Biosystems, Foster City, CA, USA). Primers were designed according to ABI guidelines and all reactions were performed using *Power* SYBR Green PCR Master Mix (Applied Biosystems), 250 nM primers (Invitrogen) and 100 ng cDNA in a 20 µL reaction volume. The following PCR protocol was used: step 1: 50°C for 2 min; step 2: 95°C for 10 min; step 3: 95°C for 15 s; step 4: 60°C for 1 min; repeat steps 3 and 4 for 40 cycles. All samples were run in quadruplicate for three independent runs and normalized against an endogenous internal control, *B2m*. PCR products were electrophoresed on a 1% agarose/TBE gel containing ethidium bromide and photographed on a Kodak Electrophoresis Documentation and Analysis System 120 Camera (Kodak, Rochester, NY, USA) to confirm amplicon size. The qRT-PCR primers used can be found in Table S1.

### Chromatin immunoprecipitation

HEK 293T cells were seeded onto 10 cm dishes and cultured to 80–90% confluency in Dulbecco's modified Eagle's medium (DMEM; GIBCO, Invitrogen) supplemented with 10% fetal bovine serum (GIBCO), 100 IU/mL penicillin and 100 µg/mL streptomycin. The cells were treated with 1% formaldehyde for 10 min at 37°C, washed twice with cold PBS containing protease inhibitors and harvested. Chromatin immunoprecipitation was carried out using the Chromatin Immunoprecipitation (ChIP) Assay Kit (Millipore) according to the manufacturer's instructions. Cell lysates were precipitated with 3 µg of either an anti-Trps1 rabbit polyclonal antibody (gift of Dr. Ramesh Shivdasani, Dana-Farber Cancer Institute, Harvard Medical School; [18]) or normal rabbit IgG (Santa Cruz Biotechnology) as a negative control. After elution, DNA was recovered using the Rapid PCR Purification System (Marligen Biosciences, Inc., Ijamsville, MD, USA). PCR reactions were performed using input, IgG-precipitated and Trps1-precipitated DNA samples, Platinum PCR SuperMix (Invitrogen) and 0.67 µM primers (Invitrogen) in a 30 µL reaction volume. The primers used for the various promoter regions as well as coding sequence negative controls can be found in Table S2. The following PCR protocol was used: step 1: 94°C for 5 min; step 2: 94°C for 45 s; step 3: 55°C for 30 s; step 4: 72°C for 1 min; repeat steps 2–4 for 36–40 cycles; step 5: 72°C for 10 min. PCR products were electrophoresed on a 1% agarose/TBE gel containing ethidium bromide and photographed on a Kodak Electrophoresis Documentation and Analysis System 120 Camera (Kodak). Positive immunoprecipitation results were confirmed in at least two independent trials.

### Promoter assays

To generate the mTrps1 expression plasmid, the open reading frame of Trps1 was amplified by PCR and subcloned into the SacI and KpnI sites of the mammalian expression vector pCXN2.1 [47]. The *hSOX9* promoter (3145 bp) was amplified by PCR from BAC clone RP11-727K24 using the following primers:

hSOX9p-F-MluI: 5′-CAAACGCGTTTCTACCTGTGTCTGAGGTC-3′

hSOX9p-R-HindIII: 5′-GACAAGCTTAGGGGTCCAGGAGATTCATA-3′

The amplified product was subcloned into the MluI and HindIII sites of the luciferase reporter vector pGL3-Basic (Promega, Madison, WI, USA). Mutated promoter reporter plasmids were generated by introducing a point mutation in select consensus GATA binding sites (WGATAR→WGATCR) using the QuikChange II XL Site-Directed Mutagenesis Kit (Agilent Technologies, Inc., Santa Clara, CA, USA) according to the manufacturer's instructions. Mutagenic primers were designed using the web-based QuikChange Primer Design Program (http://www.stratagene.com/qcprimerdesign) and can be found in Table S3.

HEK 293T cells were seeded onto 6-well dishes 24 hr before transfection. At 80% confluency, a *hSOX9* promoter reporter plasmid or pGL3 backbone vector (1 µg) were transfected into each well in combination with the mTrps1 expression plasmid or pCXN2.1 backbone vector (1 µg) using Lipofectamine 2000 (Invitrogen). A plasmid encoding a β-galactosidase reporter (0.5 µg) was also transfected for normalization of transfection efficiency. The cells were cultured for 24 hr after transfection in Opti-MEM (GIBCO, Invitrogen), harvested and lysed. Luciferase and β-galactosidase signals were measured using the Luciferase Assay System (Promega) and β-Galactosidase Enzyme Assay System with Reporter Lysis Buffer (Promega), respectively, according to the manufacturer's instructions. All assays were performed in triplicate for three independent trials.

### Blood collection and DNA extraction

Peripheral blood samples were collected from family members in EDTA-containing tubes. Genomic DNA was isolated using the Gentra Puregene Blood Kit (Qiagen Inc.) according to the manufacturer's instructions.

### Copy number variation analysis

Genomic DNA was electrophoresed on a 1% agarose/TBE gel containing ethidium bromide to ensure that approximately 90 percent of the sample was greater than 10 kb in size. Samples with an OD 260/280 ratio between 1.8–2.0 and an OD 260/230 ratio greater than 1.5 were considered pure. DNA was processed according to the manufacturer's instructions for the Affymetrix Cytogenetics Whole-Genome 2.7 M Array and the Affymetrix Genome-Wide Human SNP Array 6.0 for Cytogenetics (Affymetrix, Inc., Santa Clara, CA, USA). Briefly, approximately 100 ng of genomic DNA was denatured and amplified during a 3 hr PCR reaction. After purification, a Nanodrop spectrophotometer (Thermo Fisher Scientific, Inc.) was used to ensure a DNA concentration greater than 0.55 ng/µL and an OD 260/280 ratio between 1.8–2.0. The DNA was then fragmented into 50–300 bp fragments which were confirmed by agarose gel electrophoresis. The samples were subsequently labeled before hybridization in an Affymetrix GeneChip hybridization oven (Affymetrix). Washes and staining of the arrays with streptavidin-phycoerythrin conjugates were performed with an Affymetrix GeneChip Fluidics Station 450 (Affymetrix), and images were obtained using an Affymetrix GeneChip scanner 3000 (Affymetrix).

Quality assessments of the raw data and copy number analyses were performed with Affymetrix Chromosome Analysis Suite Version 1.0.1 software (Affymetrix). For quality control, the Median Absolute Pairwise Difference (MAPD) metric was used to estimate variability on a per-chip basis. If log_2_ ratios are distributed normally with a constant standard deviation (SD), MAPD/0.96 is equal to SD and MAPD*1.41 is equal to interquartile range. With a constant log_2_ ratio SD of 0.3, MAPD values less than 0.27 were considered acceptable for copy number analysis. In accordance with the software baseline parameters, a default diagonal weight of 0.995 was employed to minimize frequent changes in copy number. Copy number variants greater than 200 kb in length were considered significant. A pan-ethnic control reference set derived from 24 males and 24 females generated in our facility was incorporated into the analysis.

### Quantitative PCR

qPCR was performed on an ABI 7300 machine and analyzed with ABI Relative Quantification Study software (Applied Biosystems). Primers were designed according to ABI guidelines and all reactions were performed using Power SYBR Green PCR Master Mix (Applied Biosystems), 500 nM primers (Invitrogen) and 50 ng genomic DNA in a 20 µL reaction volume. The following PCR protocol was used: step 1: 50°C for 2 min; step 2: 95°C for 10 min; step 3: 95°C for 15 s; step 4: 60°C for 1 min; repeat steps 3 and 4 for 40 cycles. All samples were run in triplicate for three independent runs and normalized against an internal control, *GAPDH*. PCR products were electrophoresed on a 1% agarose/TBE gel containing ethidium bromide and photographed on a Kodak Electrophoresis Documentation and Analysis System 120 Camera (Kodak) to confirm amplicon size. The qPCR primers used can be found in Table S4.

### Fluorescent *in situ* hybridization

Lymphoblasts from peripheral patient blood samples were cultured and harvested and interphase chromosome spreads were prepared using standard cytogenetic protocols. Slides were dried at room temperature overnight, then washed in 2× saline sodium citrate (SSC) buffer at 73°C for 2 min and dehydrated through an ethanol series. Fluorescent labeled probes (Empire Genomics, Buffalo, NY, USA) were diluted to a final concentration of 40 ng/µL in hybridization buffer (Empire Genomics) and denatured at 73°C for 2 min. After probe application, slides were covered with glass coverslips and hybridized at 37°C for 16 hours in a StatSpin ThermoBrite system (Iris Sample Processing, Inc., Westwood, MA, USA). After removal of the glass coverslips, slides were placed in buffer containing 0.4× SSC and 0.3% NP-40 at 73°C for 10 s with agitation, followed by a 2 min incubation without agitation. Slides were then transferred to buffer containing 2× SSC and 0.1% NP-40 at room temperature for 1 min. Slides were dried in the dark and 10% DAPI was applied to counterstain chromosomes. Hybridized interphase chromosomes were photographed using a Nikon microscope fitted with a filter wheel and Cytovision Applied Imaging software.

## Supporting Information

Figure S1 Expression of Sox9 during vibrissa follicle morphogenesis. (A) Immunofluorescence analysis demonstrated increased Sox9 expression (red) in the suprabasal layers of the epithelial placode at E12.5. (B) Sox9 was expressed throughout the epithelial compartment of the invaginating follicle at E14.5, with the exception of the matrix. (C,D) Sox9 continued to be expressed throughout the follicle epithelium from E16.5–E18.5, with increased expression in the matrix, inner root sheath and outer root sheath layers. (E, E′) By P0 Sox9 expression became restricted to the outer root sheath cells extending along the length of the follicle. (C′,D′,E″) Faint Sox9 expression was also detected in the dermal papilla as early as E14.5 and in the dermal cells of the collagen capsule surrounding the developing vibrissae follicles. Nuclei were stained with DAPI (blue). Scale bars, 100 µm.
(TIF)

Figure S2 Colocalization of Trps1 and Shh in the vibrissa follicle. Trps1 (green) (A) and β-galactosidase (red) (B) colocalize (C) in the matrix and inner root sheath of adult *Shh^Ires-nLacZ^* vibrissae follicles as detected by immunofluorescence analyses on serial sections. Nuclei were stained with DAPI (blue). Scale bars, 100 µm.
(TIF)

Figure S3 Fluorescent *in situ* hybridization (FISH) analysis of chromosome 17q duplication orientation. Interphase chromosome spreads prepared from patient II-2 were hybridized with fluorescently-labeled probes RP11 clone 164B17 (green) and RP11 clone 831L20 (orange), which span chromosome 17q base pairs 65,334,626–65,500,838 and 66,328,117–66,543,177, respectively, according to build hg18. FISH analysis revealed one wild-type chromosome with a single hybridization signal for each probe (arrowheads) in the patient cells and one chromosome containing duplicated genetic material with two hybridization signals for each probe (arrows). The pattern of probe hybridization in the rearranged chromosome demonstrated that the 1.2 Mb duplication in patient II-2 was an inverted duplication.
(TIF)

Table S1 Primers used in qRT-PCR analyses.
(DOC)

Table S2 Primers used in chromatin immunoprecipitation experiments.
(DOC)

Table S3 Mutagenic primers used to generate plasmids for promoter assays.
(DOC)

Table S4 Primers used in qPCR analyses.
(DOC)
