# Supplementary figures and images for: Trps1 and Its Target Gene Sox9 Regulate Epithelial Proliferation in the Developing Hair Follicle and Are Associated with Hypertrichosis
Source: PLoS Genet. 2012 Nov 1;8(11):e1003002. doi: 10.1371/journal.pgen.1003002 (PMC3486859; doi:10.1371/journal.pgen.1003002)

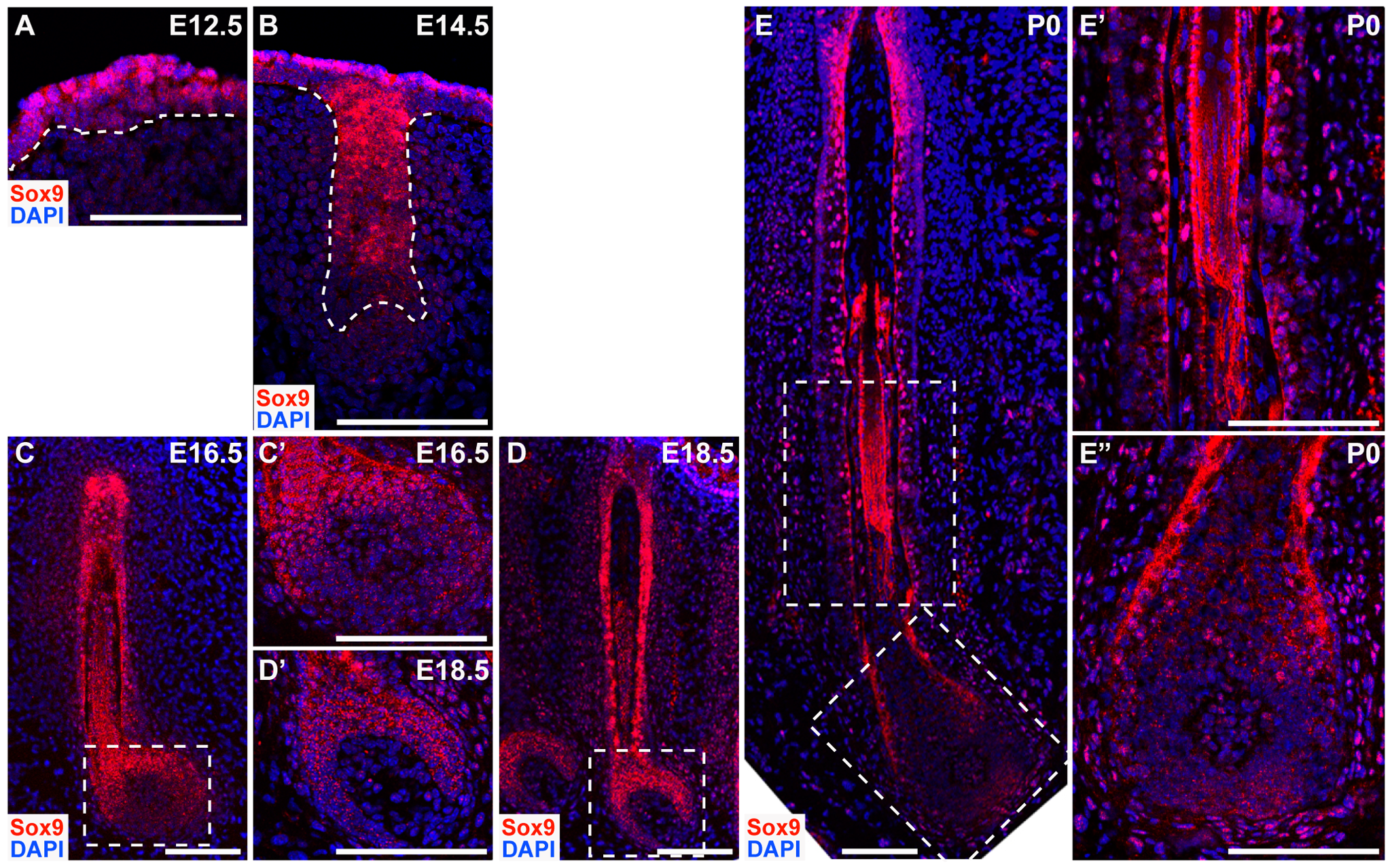

Supplement: Figure S1 — Expression of Sox9 during vibrissa follicle morphogenesis. (A) Immunofluorescence analysis demonstrated increased Sox9 expression (red) in the suprabasal layers of the epithelial placode at E12.5. (B) Sox9 was expressed throughout the epithelial compartment of the invaginating follicle at E14.5, with the exception of the matrix. (C,D) Sox9 continued to be expressed throughout the follicle epithelium from E16.5–E18.5, with increased expression in the matrix, inner root sheath and outer root sheath layers. (E, E′) By P0 Sox9 expression became restricted to the outer root sheath cells extending along the length of the follicle. (C′,D′,E″) Faint Sox9 expression was also detected in the dermal papilla as early as E14.5 and in the dermal cells of the collagen capsule surrounding the developing vibrissae follicles. Nuclei were stained with DAPI (blue). Scale bars, 100 µm. (TIF) [file pgen.1003002.s001.tif]

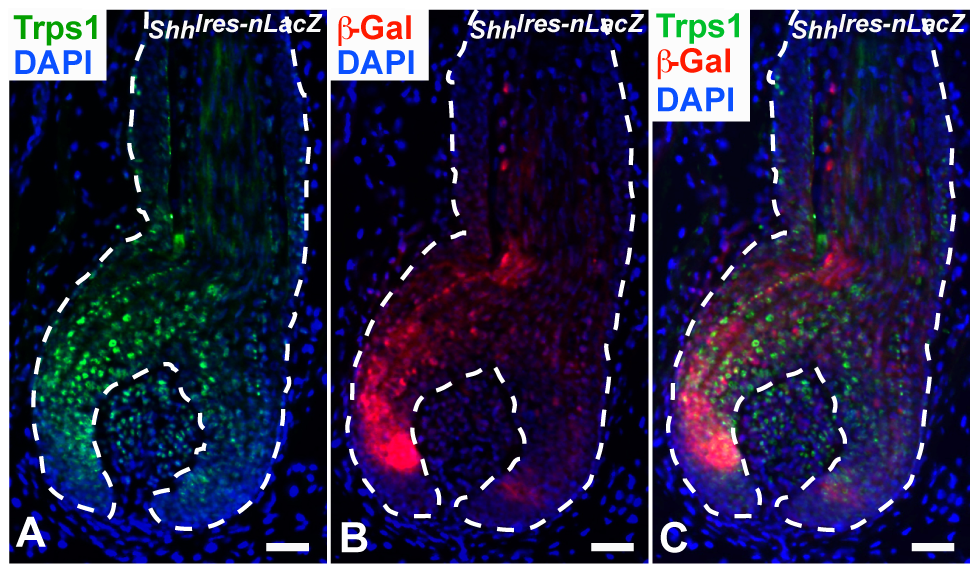

Supplement: Figure S2 — Colocalization of Trps1 and Shh in the vibrissa follicle. Trps1 (green) (A) and β-galactosidase (red) (B) colocalize (C) in the matrix and inner root sheath of adult ShhIres-nLacZ vibrissae follicles as detected by immunofluorescence analyses on serial sections. Nuclei were stained with DAPI (blue). Scale bars, 100 µm. (TIF) [file pgen.1003002.s002.tif]

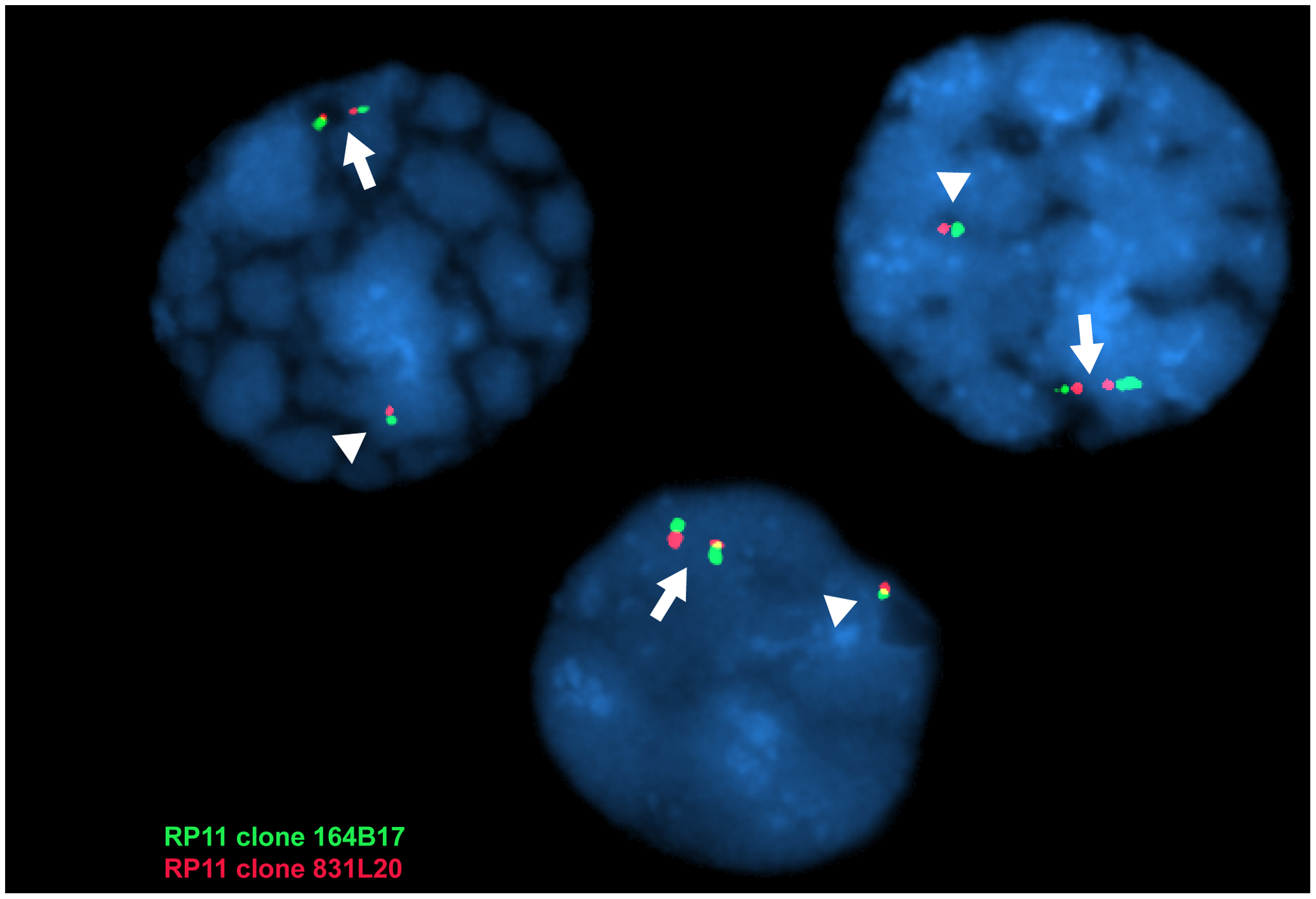

Supplement: Figure S3 — Fluorescent in situ hybridization (FISH) analysis of chromosome 17q duplication orientation. Interphase chromosome spreads prepared from patient II-2 were hybridized with fluorescently-labeled probes RP11 clone 164B17 (green) and RP11 clone 831L20 (orange), which span chromosome 17q base pairs 65,334,626–65,500,838 and 66,328,117–66,543,177, respectively, according to build hg18. FISH analysis revealed one wild-type chromosome with a single hybridization signal for each probe (arrowheads) in the patient cells and one chromosome containing duplicated genetic material with two hybridization signals for each probe (arrows). The pattern of probe hybridization in the rearranged chromosome demonstrated that the 1.2 Mb duplication in patient II-2 was an inverted duplication. (TIF) [file pgen.1003002.s003.tif]
