# Supplementary material for: Trps1 and Its Target Gene Sox9 Regulate Epithelial Proliferation in the Developing Hair Follicle and Are Associated with Hypertrichosis
Source: PLoS Genet. 2012 Nov 1;8(11):e1003002. doi: 10.1371/journal.pgen.1003002 (PMC3486859; doi:10.1371/journal.pgen.1003002)
Supplement: Table S1 — Primers used in qRT-PCR analyses. (DOC) [file pgen.1003002.s004.doc]

**Table S1. Primers used in qRT-PCR analyses.**

| **Transcript** | **Forward Primer (5’ to 3’)** | **Reverse Primer (5’ to 3’)** |
| --- | --- | --- |
| *mB2m* | ACTGACCGGCCTGTATGCTA | TGAAGGACATATCTGACATCTCTA |
| *mSox9* | GGAACAGACTCACATCTCTC | GATCAACTTTGCCAGCTTGC |
