# Supplementary material for: Trps1 and Its Target Gene Sox9 Regulate Epithelial Proliferation in the Developing Hair Follicle and Are Associated with Hypertrichosis
Source: PLoS Genet. 2012 Nov 1;8(11):e1003002. doi: 10.1371/journal.pgen.1003002 (PMC3486859; doi:10.1371/journal.pgen.1003002)
Supplement: Table S2 — Primers used in chromatin immunoprecipitation experiments. (DOC) [file pgen.1003002.s005.doc]

**Table S2. Primers used in chromatin immunoprecipitation experiments.**

| **Region** | **Position Relative to Exon 1** | **Forward Primer (5’ to 3’)** | **Reverse Primer (5’ to 3’)** |
| --- | --- | --- | --- |
| *hSOX9* pR1 | -2604 to -2450 | AAATGCAGGACCGATCCACT | AAGAGCACTTGAGATGCCAG |
| *hSOX9* pR2 | -1526 to -1376 | CTCCCGGAAGGACATTGATT | ACCCTTTTGGGCTCTTGCAA |
| *hSOX9* pR3 | -1314 to -1164 | TGAAAGCACAGAACCCGCAA | GCATTGGTGGTGTCTCTCAT |
| *hSOX9* pR4 | -1014 to -866 | TGGTGCCCATTTGTTTGGTC | TCAAAAACGTCAGCCGAGTC |
| *hSOX9* pR5 | -650 to -496 | TGCAAAAGCGCAGCAGAATC | CCTGCCTGCAAAAGTGCTTA |
| *hSOX9* CDS | Exon 1 | ATGAAGATGACCGACGAGCA | TCGCTCTCCTTCTTCAGATC |

Abbreviations: pR, promoter region; CDS, coding sequence.
