# Supplementary material for: Trps1 and Its Target Gene Sox9 Regulate Epithelial Proliferation in the Developing Hair Follicle and Are Associated with Hypertrichosis
Source: PLoS Genet. 2012 Nov 1;8(11):e1003002. doi: 10.1371/journal.pgen.1003002 (PMC3486859; doi:10.1371/journal.pgen.1003002)
Supplement: Table S3 — Mutagenic primers used to generate plasmids for promoter assays. (DOC) [file pgen.1003002.s006.doc]

**Table S3. Mutagenic primers used to generate plasmids for promoter assays.**

| **Region** | **Forward Primer (5’ to 3’)** | **Reverse Primer (5’ to 3’)** |
| --- | --- | --- |
| *hSOX9* pR1* | CCAAATTCTGCGCAGACTAGAACGGCTGGCATC | GATGCCAGCCGTTCTAGTCTGCGCAGAATTTGG |
| *hSOX9* pR3a* | AAGCGACCAAGACTTTTCTTCTAGCCCAGAGCAGAT | ATCTGCTCTGGGCTAGAAGAAAAGTCTTGGTCGCTT |
| *hSOX9* pR3b* | CTATCCCAGAGCAGATCGCTCCGCACTTACC | GGTAAGTGCGGAGCGATCTGCTCTGGGATAG |
| *hSOX9* pR5a* | GAACCCGAGCATGTTAATCTAGTTATATGGATTATTACGGAGG | CCTCCGTAATAATCCATATAACTAGATTAACATGCTCGGGTTC |
| *hSOX9* pR5b* | AGTCACCAAAACATTTGCTTCAAAAGACTAGTTCTAAGCACTTTTGC | GCAAAAGTGCTTAGAACTAGTCTTTTGAAGCAAATGTTTTGGTGACT |

Abbreviation: pR, promoter region.
