# Supplementary material for: Trps1 and Its Target Gene Sox9 Regulate Epithelial Proliferation in the Developing Hair Follicle and Are Associated with Hypertrichosis
Source: PLoS Genet. 2012 Nov 1;8(11):e1003002. doi: 10.1371/journal.pgen.1003002 (PMC3486859; doi:10.1371/journal.pgen.1003002)
Supplement: Table S4 — Primers used in qPCR analyses. (DOC) [file pgen.1003002.s007.doc]

**Table S4. Primers used in qPCR analyses.**

| **Amplicon** | **Chromosome 17 Position** | **Forward Primer (5’ to 3’)** | **Reverse Primer (5’ to 3’)** |
| --- | --- | --- | --- |
| 1 | 63778770-63778994 | GTCTCTTTGCAACACTGGGA | AGGCAAACAGAGACACAGTC |
| 2 | 64549834-64549940 | CTCTAATGCATCACTTGGCT | GAGCTTTATGGCCAGAGATG |
| 3 | 66249690-66249851 | GACAATAATGAGCATCTGTGG | GCAAACTATACGAGCTGTGA |
| 4 | 67380284-67380441 | CAGACCTCAAGCTGGAAATG | GCATCTTGACTGCTTTCGTG |
| *hGAPDH* | -- | ATGGACACGCTCCCCTGACT | GAAAGGTGGGAGCCTCAGTC |

Chromosome 17 position according to build hg18.
